# Serum electrolyte levels in relation to macrovascular complications in Chinese patients with diabetes mellitus

**DOI:** 10.1186/1475-2840-12-146

**Published:** 2013-10-10

**Authors:** Shenqi Wang, Xuhong Hou, Yu Liu, Huijuan Lu, Li Wei, Yuqian Bao, Weiping Jia

**Affiliations:** 1 Department of Endocrinology and Metabolism, Shanghai Jiao Tong University Affiliated Sixth People’s Hospital, Shanghai Diabetes Institute, Shanghai Clinical Center for Diabetes, 600 Yishan Road, Shanghai, 200233, China

**Keywords:** Electrolytes, Macrovascular complications, Normal glucose regulation, Impaired glucose regulation, Diabetes mellitus

## Abstract

**Background:**

The prevalence of diabetes in China is increasing rapidly. However, scarce data are available on serum electrolyte levels in Chinese adults with diabetes, especially in those with cardiovascular complications. This study measured serum electrolyte levels and examined their relationship with macrovascular complications in Chinese adults with diabetes.

**Methods:**

The three gender- and age-matched groups were enrolled into this analysis, which were 1,170 subjects with normal glucose regulation (NGR), 389 with impaired glucose regulation (IGR) and 343 with diabetes. Fasting plasma glucose (FPG), 2-hour post-load plasma glucose (2hPG), glycosylated hemoglobin A1c (HbA1c) and serum electrolyte levels were measured. Data collection included ankle brachial index results.

**Results:**

Serum sodium and magnesium levels in the diabetes group were significantly decreased compared to the NGR group (sodium: 141.0 ± 2.4 vs. 142.1 ± 2.0 mmol/l; magnesium: 0.88 ± 0.08 vs. 0.91 ± 0.07 mmol/l, all *P* < 0.01), while the serum calcium level was significantly increased (2.36 ± 0.11 vs. 2.33 ± 0.09 mmol/l, *P* < 0.01). Multiple linear regression showed that serum sodium and magnesium levels in the diabetes group were negatively correlated with FPG, 2hPG and HbA1c (sodium: Std β = −0.35, -0.19, -0.25; magnesium: Std β = −0.29, -0.17, -0.34, all *P* < 0.01), while the serum calcium level was positively correlated with HbA1c (Std β = 0.17, *P* < 0.05). In diabetic subjects, serum sodium, magnesium and potassium levels were decreased in the subjects with the elevation of estimated glomerular filtration rates (*P* < 0.05). ANCOVA analysis suggested that serum magnesium level in subjects with diabetic macrovascular complications was significantly decreased compared with diabetic subjects without macrovascular complications after the effect of some possible confounding being removed (*P* < 0.05).

**Conclusions:**

Serum sodium and magnesium levels were decreased in Chinese subjects with diabetes, while the observed increase in calcium level correlated with increasing glucose level. Diabetic patients with macrovascular complications had lower serum magnesium level than those with no macrovascular complications.

## Introduction

The electrolytes in serum include sodium (Na^+^), potassium (K^+^), calcium (Ca^2+^) and magnesium (Mg^2+^) [1]. These electrolytes play an important role in intermediary metabolism and cellular function, including enzyme activities and electrical gradients [2]. Serum concentrations of electrolytes have been shown to change with plasma glucose levels [3]. Disturbances in the levels of some electrolytes are associated with diabetes mellitus (DM) [4-7]. In addition, hypomagnesemia and diuretic-associated hypokalemia may lead to a higher incidence of DM [8,9], mild electrolyte changes such as low Mg^2+^ levels can predict mortality in type 2 DM [10] and oral magnesium supplementation reduces fasting plasma glucose levels in DM patients [11]. The Atherosclerosis Risk in Communities (ARIC) study has shown an association between low serum magnesium level and an increased risk of ischemic stroke in African Americans and Caucasians [12].

The prevalence of DM in China is increasing rapidly [13]. However, scant data are available on serum electrolyte levels in Chinese adults with diabetes, especially in those with cardiovascular complications, such as cardiovascular disease (CVD) events and peripheral arterial disease (PAD) [14]. In this study, we measured serum electrolyte levels in Chinese subjects with diabetes, to investigate the relationship between electrolytes and glucose levels, and we focus on examining the differences in serum electrolytes in diabetic patients with or without macrovascular complications.

## Methods

### Study population

**Figure 1:**
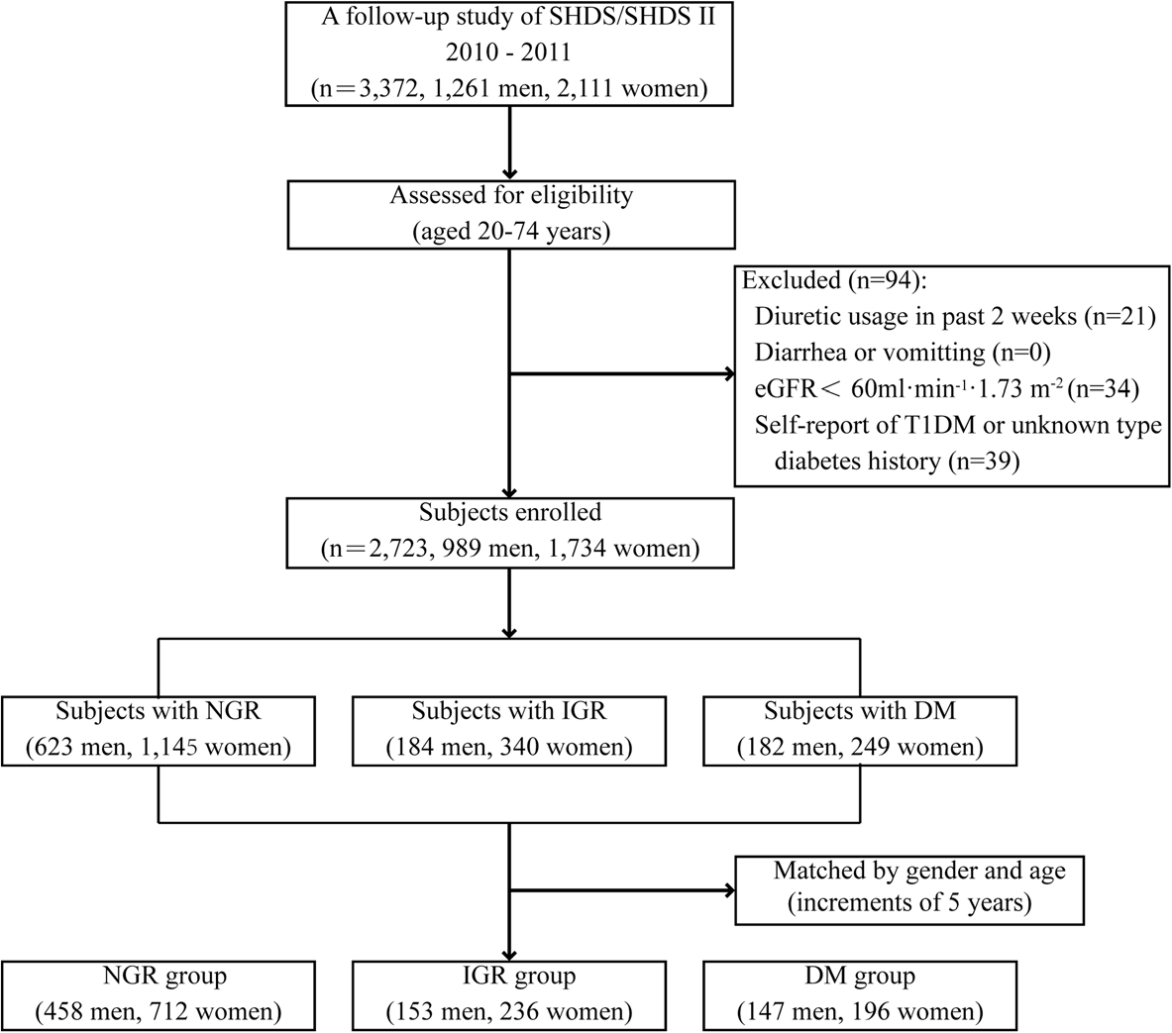
Flow diagram of subject recruitment.

A gender- and age- matched case–control analysis was performed based on data obtained from the follow-up study of the Shanghai Diabetes Study (SHDS) and SHDS II [15,16]. As shown in Figure 1, this follow-up study was conducted from August 2010 to December 2011 in the Caoyang, Anting, and Huayang communities in Shanghai, China. Subjects enrolled in the analysis met the following inclusion criteria: aged 20 years or above and under 75 years, with complete serum electrolytes and fasting glucose data. Exclusion criteria included subjects once taking diuretics in the 2 weeks before being interviewed (n = 21), persistent diarrhea or vomiting (n = 0), severe renal insufficiency, which was determined using an estimated glomerular filtration rate (eGFR) <60 ml · min^-1^ · 1.73 m^-2^ (n = 34) and any self-report of type 1 diabetes mellitus (T1DM) or a history of diabetes of an unknown type (n = 39). In total, 2,723 subjects were enrolled in the study and were grouped according to their glucose tolerance. They were matched (the number ratio of subjects in each group nearly 3:1:1) according to their gender and age (5-year intervals). Then, a normal glucose regulation (NGR) group (n = 1,170), an impaired glucose regulation (IGR) group (n = 389) and a DM group (n = 343) were formed.

This study was approved by the institutional review board of Shanghai Jiao Tong University Affiliated Sixth People’s Hospital in accordance with the principle of the Helsinki Declaration II. Written informed consent was obtained from each subject before the survey.

### Laboratory assessment

Subjects were invited to the local hospital between 6 and 8 am following an overnight 8–10 h fast. Venous blood samples were collected at 0 and 120 min following a 75-g oral glucose tolerance test (OGTT) for subjects without self-reported diabetes, or after the consumption of a steamed bun containing approximately 80 g of complex carbohydrates for subjects with self-reported histories of DM. Fasting blood samples were used to obtain serum electrolyte levels and other biochemical analyses. Plasma glucose, electrolytes, creatinine levels (SCr) and lipid profiles were determined with an autoanalyzer (Hitachi 7600 analyzer, Hitachi, Japan). The inter- and intra-assay coefficients of variation for Na^+^, K^+^, Ca^2+^, Mg^2+^ were 0.77% and 1.13%; 1.01% and 1.22%; 1.80% and 3.00%; and 1.15% and 1.92% respectively. Serum glycosylated hemoglobin (HbA1c) was measured using high-performance liquid chromatography. The eGFR was assessed using the following formula: eGFR (ml · min^-1^ · 1.73 m^-2^) = 186 × SCr (mg · dl^-1^)^-1.154^ × age^-0.203^ (for women × 0.742) [17].

### Interviews and measurements

Demographic and disease history information was collected again using a standardized questionnaire during this follow-up study. The anthropometric indices of height and weight were measured while subjects were barefoot and wearing light clothing. The waist circumference (WC) was measured at the point between the costal margin and iliac crests. Body mass index (BMI) was calculated as weight in kilograms divided by height in meters squared [15]. A blood pressure measurement was taken twice using a mercury sphygmomanometer and the results averaged [18]. A Doppler probe (Nicolet VersaLab SE) was used to calculate the ankle brachial index (ABI), which is equal to the highest measured arterial pressure in the ankle or foot divided the pressure in the arm on each side [19].

### Definitions and diagnostic criteria

DM was defined as a fasting plasma glucose (FPG) ≥7.0 mmol/l and/or a 2-hour post-load plasma glucose (2hPG) ≥11.1 mmol/l, or on diabetic medication treatment for type 2 diabetes (T2DM). IGR was defined by an impaired fasting glucose (6.1 mmol/l < FPG <7.0 mmol/l and 2hPG <7.8 mmol/l), impaired glucose tolerance (FPG < 6.1 mmol/l and 7.8 mmol/l < 2hPG < 11.1 mmol/l), and impaired fasting glucose with impaired glucose tolerance (6.1 mmol/l < FPG <7.0 mmol/l and 7.8 mmol/l < 2hPG <11.1 mmol/l) in the follow-up [20]. Normal serum electrolyte reference ranges were used as follows: 137 mmol/l ≤ Na^+^ ≤ 145 mmol/l, 3.5 mmol/l ≤ K^+^ ≤ 5.1 mmol/l, 2.08 mmol/l ≤ Ca^2+^ ≤ 2.60 mmol/l, 0.65 mmol/l ≤ Mg^2+^ ≤ 1.05 mmol/l. Hypertension was defined as a blood pressure of >140/90 mmHg or the use of antihypertensive medications [21]. Dyslipidemia was defined as a serum triglyceride (TG) level ≥1.7 mmol/L or a high density lipoprotein cholesterol (HDL-C) level <0.9 mmol/L in men and <1.0 mmol/L in women [22].

Diabetic macrovascular complications in this study included any patient with a history of CVD events or PAD. Subjects were defined as having had CVD events if a history of coronary heart disease (CHD) or stroke was evident. Various non-fatal CVDs were determined according to the self-reports of patients, which were confirmed by their medical records. The definition of CHD included a history of hospitalization for myocardial infarction, a surgical history of coronary balloon angioplasty, coronary stent implantation or coronary artery bypass. Stroke was defined as a history of language or physical dysfunction that continued for more than 24 h, and ischemic or hemorrhagic stroke was diagnosed using imaging examinations (computed tomography or magnetic resonance imaging) [23].

According to the current guidelines proposed by the American Heart Association (AHA), PAD was identified as an ABI <0.90 in either leg. Those with an ABI >1.3 were excluded from the analysis to avoid those with significant medial artery layer calcification, which is independent of atherosclerotic plaques [24]; other individuals were considered as non-PAD [19].

### Statistical methods

Summarized data are shown as mean ± standard deviation or as frequencies (%). One-way analysis of variance (one-way ANOVA) was used to compare the mean differences and the Pearson’s chi-square test was used to compare the proportional differences in demographic characteristics and serum electrolyte levels among the NGR, IGR and DM groups, or among the mildly decreased eGFR, normal eGFR and elevated eGFR groups. The linear trends of electrolyte levels within the groups were tested. Multiple linear regression analyses were used to determine the relationships between electrolytes and glucose levels. Analysis of covariance (ANCOVA) was used to compare the mean differences in electrolyte levels after adjustment by possible confounding factors between DM subjects with macrovascular complications and those without macrovascular complications. SPSS for windows (version 17.0, SPSS, Chicago, IL, USA) was used to perform the statistical analyses. The significance level was *P* value <0.05, and the results were adjusted with the Bonferroni correction for multiple testing.

## Results

### Characteristics of the study population

The gender and age distributions of all subjects prior to matching, and of the remaining unmatched subjects were shown in Table 1. After matching, an overall total of 1,902 subjects (758 men, 1,144 women) with a mean age of 57.4 ± 8.8 years participated in this study. As shown in Table 2, among the NGR, IGR and DM groups, there were no significant differences in gender or age. There were significant differences in BMI, WC, systolic blood pressure (SBP), diastolic blood pressure (DBP), total cholesterol (TC), TG, HDL-C, SCr, eGFR, FPG, 2hPG, and HbA1c. In the DM group, 231 (65.3%) subjects had hypertension, 173 (48.9%) had dyslipidemia and 37 (10.5%) had a history of CVD events. These proportions were significantly higher in the DM group than in the NGR group. There was no significance in the proportion of individuals affected by PAD among the three groups.

**Table 1:** Gender and age distributions of all subjects prior to matching, and those of the unmatched and matched subjects

	**NGR (Group I)**	**IGR (Group II)**	**DM (Group III)**
Before matching			
n (men/women)	1768 (623/1145)	524 (184/340)	431 (182/249)
Age (years)	52.6 ± 11.5	57.2 ± 10.2	60.0 ± 9.2
Unmatched			
n (men/women)	598 (165/433)	135 (31/104)	88 (35/53)
Age (years)	43.8 ± 11.1	56.5 ± 12.8	67.2 ± 7.4
Matched			
n (men/women)	1170 (458/712)	389 (153/236)	343 (147/196)
Age (years)	57.1 ± 8.7	57.5 ± 9.1	58.1 ± 8.6

Descriptive values are expressed as the frequency or mean ± standard deviation.

**Table 2:** Characteristics of the subjects grouped according to glucose tolerance status

**Characteristics**	**NGR (Group I)** **(n = 1170)**	**IGR (Group II) (n = 389)**	**DM (Group III) (n = 343)**	***P*-value**			
				**All**	**I vs. II**	**II vs. III**	**I vs. III**
Age (years)	57.1 ± 8.7	57.5 ± 9.1	58.1 ± 8.6	0.19	1.000	1.000	0.216
BMI (Kg · m^-2^)	23.3 ± 3.6	24.6 ± 3.5	25.2 ± 4.0	<0.01	<0.001	0.120	<0.001
WC (cm)	81.1 ± 9.4	84.8 ± 9.2	86.8 ± 9.1	<0.01	<0.001	0.011	<0.001
SBP (mmHg)	124 ± 16	129 ± 18	132 ± 17	<0.01	<0.001	0.017	<0.001
DBP (mmHg)	79 ± 10	81 ± 10	83 ± 9	<0.01	0.028	0.015	<0.001
TC (mmol/L)	5.17 ± 0.95	5.32 ± 0.97	5.39 ± 1.00	<0.01	0.028	0.956	0.001
TG (mmol · L^-1^)	1.45 ± 1.02	1.79 ± 1.17	2.15 ± 2.04	<0.01	<0.001	<0.001	<0.001
HDL-C (mmol · L^-1^)	1.44 ± 0.34	1.34 ± 0.31	1.27 ± 0.29	<0.01	<0.001	0.011	<0.001
SCr (umol · L^-1^)	66.0 ± 12.7	65.5 ± 13.8	64.0 ± 14.8	0.04	1.000	0.371	0.041
eGFR (ml · min^-1^ · 1.73 m^-2^)	99.4 ± 16.9	100.9 ± 18.6	105.6 ± 22.4	<0.01	0.515	0.002	<0.001
FPG (mmol · L^-1^)	5.22 ± 0.45	5.85 ± 0.61	8.30 ± 2.62	<0.01	<0.001	<0.001	<0.001
2hPG (mmol · L^-1^)	5.77 ± 1.11	8.15 ± 1.50	14.63 ± 5.03	<0.01	<0.001	<0.001	<0.001
HbA1c (%)	5.5 ± 0.4	5.7 ± 0.5	7.2 ± 1.6	<0.01	<0.001	<0.001	<0.001
Hypertension (%)	462 (39.5)	212 (54.5)	231 (65.3)	<0.01	<0.001	0.003	<0.001
Dyslipidemia (%)	305 (26.1)	160 (41.1)	173 (48.9)	<0.01	<0.001	0.033	<0.001
CVD (%)	66 (5.6)	34 (8.7)	37 (10.5)	<0.01	0.031	0.350	0.001
PAD (%)	38 (3.3)	19 (4.9)	15 (4.2)	0.23	0.323	0.334	0.150

Descriptive values are expressed as the mean ± standard deviation or frequency (%). Differences are assessed using the one-way ANOVA test for means and by the chi-square test for proportions. The Bonferroni correction is applied for the multiple testing.

*Abbreviations*: *NGR* Normal glucose regulation, *IGR* Impaired glucose regulation, *DM* Diabetes mellitus, *BMI* Body mass index, *WC* Waist circumstance, *SBP* Systolic blood pressure, *DBP* Diastolic blood pressure, *PAD* Peripheral arterial disease, *TC* Total cholesterol, *TG* Triglyceride, *HDL* High density lipoprotein cholesterol, *FPG* Fasting plasma glucose, *2hPG* 2-hour post-load plasma glucose, *HbA1c* Glycosylated hemoglobin.

The study included 343 subjects with diabetes (147 men, 196 women) with a mean age of 58.1 ± 8.6 years. There were 178 self-reported T2DM subjects (80 men, 98 women) in them. In the self-reported T2DM subjects, the mean diabetes duration was 9.1 ± 6.2 years and 31 (17.4%) subjects claimed to be currently receiving insulin therapy. Medications that were taken at the time of the study by individuals in the NGR, IGR and DM groups were also shown in Table 3.

**Table 3:** Current medication among the groups

**Medications**	**NGR (Group I) (n = 1170)**	**IGR (Group II) (n = 389)**	**DM (Group III) (n = 343)**
Antihypertensive drugs			
Diuretic (%)	0 (0.0)	0 (0.0)	0 (0.0)
ARB (%)	77 (6.6)	25 (6.4)	46 (13.4)
ACEI (%)	27 (2.3)	16 (4.1)	23 (6.7)
β-blocker (%)	12 (1.0)	10 (2.6)	5 (1.5)
CCB (%)	86 (7.4)	53 (13.6)	59 (17.2)
Others (%)	36 (3.1)	24 (6.2)	10 (2.9)
Antidiabetic drugs			Self-reported T2DM (n = 178)
Insulin (%)			31 (17.4)
Oral hypoglycemic drugs			
Metformin (%)			61 (34.3)
Sulphonylurea (%)			41 (23.0)
Glinide (%)			26 (14.6)
Thiazolidinedione (%)			10 (5.6)
α-Glucosidase inhibitor (%)			24 (13.5)
Others (%)			3 (1.7)

Descriptive values are expressed as frequency (%).

*Abbreviations*: *ARB* Angiotensin II receptor blocker, *ACEI* Angiotensin-converting enzyme inhibitor, *CCB* Calcium channel blocker.

### Serum electrolyte levels among the three groups

Among the NGR, IGR and DM groups, 9 (0.8%), 4 (1.0%) and 14 (4.1%) subjects had hyponatremia (*P* < 0.01), while 55 (4.7%), 17 (4.4%), and 10 (2.9%) subjects had hypernatremia (*P* = 0.36); 14 (1.2%), 11 (2.8%) and 2 (0.6%) had hypokalemia (*P* = 0.02), while 17 (1.5%), 3 (0.5%) and 4 (1.2%) subjects had hyperkalemia (*P* = 0.57); 6 (0.5%), 0 (0.0%) and 4 (1.2%) had hypocalcemia (*P* = 0.09), while 3 (0.3%), 0 (0.0%) and 3 (0.9%) subjects had hypercalcemia (*P* = 0.09); 0 (0.0%), 0 (0.0%) and 2 (0.6%) had hypomagnesemia (*P* = 0.01), while 26 (2.2%), 11 (2.8%) and 3 (0.9%) subjects had hypermagnesemia (*P* = 0.16).

As shown in Table 4, when compared to the NGR group, serum levels of Na^+^ and Mg^2+^ were significantly decreased (*P* < 0.01), while Ca^2+^ level was significantly increased for both genders in the DM group (*P* < 0.01). Serum Na^+^, Mg^2+^ and Ca^2+^ levels showed linear trends in the DM, IGR and NGR groups (*P* < 0.01).

**Table 4:** Serum electrolyte levels of subjects among the groups

**Electrolyte levels**	**NGR (Group I)**	**IGR (Group II)**	**DM (Group III)**	***P*-value**				
				**All**	**I vs. II**	**II vs. III**	**I vs. III**	**Linear trend**
Overall	n = 1170	n = 389	n = 343					
Na^+^ (mmol/L)	142.1 ± 2.0	142.1 ± 2.0	141.0 ± 2.4	<0.01	1.000	<0.001	<0.001	<0.01
K^+^ (mmol/L)	4.21 ± 0.37	4.17 ± 0.38	4.26 ± 0.37	0.01	0.188	0.007	0.172	0.25
Ca^2+^ (mmol/L)	2.33 ± 0.09	2.34 ± 0.09	2.36 ± 0.11	<0.01	0.110	0.034	<0.001	<0.01
Mg^2+^ (mmol/L)	0.91 ± 0.07	0.92 ± 0.08	0.88 ± 0.08	<0.01	0.643	<0.001	<0.001	<0.01
Men	n = 458	n = 153	n = 147					
Na^+^ (mmol/L)	142.0 ± 2.0	142.3 ± 1.9	141.0 ± 2.2	<0.01	0.501	<0.001	<0.001	<0.01
K^+^ (mmol/L)	4.22 ± 0.39	4.14 ± 0.38	4.26 ± 0.40	0.04	0.128	0.040	0.930	0.71
Ca^2+^ (mmol/L)	2.32 ± 0.09	2.32 ± 0.09	2.34 ± 0.10	<0.01	1.000	0.121	0.002	<0.01
Mg^2+^ (mmol/L)	0.92 ± 0.07	0.92 ± 0.08	0.89 ± 0.08	<0.01	1.000	<0.001	<0.001	<0.01
Women	n = 712	n = 236	n = 196					
Na^+^ (mmol/L)	142.1 ± 2.0	141.9 ± 2.1	141.1 ± 2.5	<0.01	1.000	<0.001	<0.001	<0.01
K^+^ (mmol/L)	4.21 ± 0.36	4.19 ± 0.37	4.26 ± 0.34	0.14	1.000	0.169	0.309	0.22
Ca^2+^ (mmol/L)	2.34 ± 0.09	2.35 ± 0.09	2.37 ± 0.11	<0.01	0.143	0.217	0.001	<0.01
Mg^2+^ (mmol/L)	0.91 ± 0.07	0.92 ± 0.08	0.87 ± 0.09	<0.01	1.000	<0.001	<0.001	<0.01

Descriptive values are expressed as the mean ± standard deviation. Differences are assessed using the one-way ANOVA. Bonferroni correction is applied for multiple testing. Linear trends of electrolyte levels within groups were tested.

*Abbreviations*: *NGR* Normal glucose regulation, *IGR* Impaired glucose regulation, *DM* Diabetes mellitus, *Na* Sodium, *K* Potassium, *Ca* Calcium, *Mg*, Magnesium.

### Serum electrolyte levels in DM subjects with different eGFRs

As shown in Table 5, DM subjects were divided into mildly decreased eGFR (eGFR 60 to 89 ml · min^-^ · 1.73 m^-2^), normal eGFR (eGFR 90 to 119 ml · min^-^ · 1.73 m^-2^) and elevated eGFR groups (eGFR over 120 ml · min^-^ · 1.73 m^-2^). Serum Na^+^, Mg^2+^ and K^+^ levels were lowest in the elevated eGFR group (*P* < 0.01) and showed decreasing trends from the mildly decreased eGFR group, normal eGFR group to the elevated eGFR group (*P* < 0.01).

**Table 5:** Serum electrolyte levels in DM subjects stratified by eGFR.

**Electrolyte levels**	**eGFR (ml · min**^**-**^ **· 1.73 m**^**-2**^**)**			***P*-value**				
	**Mildly decreased (Group I) (n = 81)**	**Normal (Group II)** **(n = 186)**	**Elevated (Group III) (n = 76)**	**All**	**I vs. II**	**II vs. III**	**I vs. III**	**Linear trend**
Na^+^ (mmol/L)	141.8 ± 2.1	141.1 ± 2.5	140.1 ± 2.1	<0.01	0.114	0.006	<0.001	<0.01
K^+^ (mmol/L)	4.36 ± 0.38	4.23 ± 0.36	4.22 ± 0.35	0.02	0.022	1.000	0.052	0.02
Ca^2+^ (mmol/L)	2.36 ± 0.11	2.35 ± 0.11	2.36 ± 0.09	0.82	1.000	1.000	1.000	0.90
Mg^2+^ (mmol/L)	0.90 ± 0.08	0.88 ± 0.08	0.84 ± 0.10	<0.01	0.308	0.003	<0.001	<0.01

Descriptive values are expressed as the mean ± standard deviation. Differences are assessed using one-way ANOVA. Bonferroni correction is applied for multiple testing. Linear trends of electrolyte levels within groups were tested.

*Abbreviations*: *eGFR* Estimated glomerular filtration rate, *NGR* Normal glucose regulation, *IGR* Impaired glucose regulation, *DM* Diabetes mellitus, *Na* Sodium, *K* Potassium, *Ca* Calcium, *Mg* Magnesium.

### Correlation of electrolytes and glucose levels in subjects with DM

As shown in Table 6, serum Na^+^ and Mg^2+^ levels were negatively correlated with FPG (Pearson’s r = −0.35 and −0.29, respectively), 2hPG (Pearson’s r = −0.19 and −0.17, respectively) and HbA1c (Pearson’s r = −0.25 and −0.34, respectively; all *P* < 0.01), while serum Ca^2+^ level was positively correlated with HbA1c (Pearson’s r = 0.17; *P* < 0.05) after adjustment for co-variables, including gender, age, BMI, SBP, DBP, TG, HDL and eGFR levels.

**Table 6:** Multiple linear regression analyses of the associations between electrolyte levels and glucose levels in DM subjects

**Dependent variables**	**Independent variables**	**Std β**	**β**	**SE**	***P*-value**	**R**^**2**^
Na^+^	FPG (mmol/L)	−0.35	−0.319	0.049	<0.01	0.23
(mmol/L)	2hPG (mmol/L)	−0.19	−0.093	0.026	<0.01	0.16
	HbA1c (%)	−0.25	−0.386	0.086	<0.01	0.18
K^+^	FPG (mmol/L)	0.01	0.001	0.008	0.99	0.04
(mmol/L)	2hPG (mmol/L)	0.03	0.002	0.004	0.63	0.04
	HbA1c (%)	0.08	0.018	0.014	0.21	0.05
Ca^2+^	FPG (mmol/L)	0.11	0.004	0.002	0.07	0.04
(mmol/L)	2hPG (mmol/L)	0.09	0.002	0.001	0.14	0.04
	HbA1c (%)	0.17	0.011	0.004	<0.01	0.06
Mg^2+^	FPG (mmol/L)	−0.29	−0.009	0.002	<0.01	0.17
(mmol/L)	2hPG (mmol/L)	−0.17	−0.003	0.001	<0.01	0.13
	HbA1c (%)	−0.34	−0.018	0.003	<0.01	0.20

Linear regression analysis, where electrolyte levels is used as the dependent variable, FPG, 2hPG, HbA1c are the independent variables, and gender, age, BMI, SBP, DBP, TG, HDL and eGFR levels are the co-variables, using the Enter method.

*Abbreviations*: *FPG* Fasting plasma glucose, *2hPG* 2-hour post-load plasma glucose, *HbA1c* Glycosylated hemoglobin, *Na* Sodium, *K* Potassium, *Ca* Calcium, *Mg* Magnesium.

### Serum electrolyte levels in subjects with and without DM macrovascular complications

As shown in Table 7, only serum Mg^2+^ level was significantly lower in DM subjects with macrovascular complications compared to those without macrovascular complications. The mean value of serum Mg^2+^ level was 0.86 ± 0.10 mmol/L and 0.87 ± 0.08 mmol/L, respectively (*P* < 0.05). In detail, the mean value of serum Mg^2+^ level was 0.85 ± 0.11 mmol/L and 0.88 ± 0.08 mmol/L in subjects with and without CVD events, respectively, and 0.85 ± 0.10 mmol/L and 0.87 ± 0.08 mmol/L in subjects with and without PAD, respectively.

**Table 7:** Serum electrolyte levels in DM subjects with and without macrovascular complications

**Electrolyte levels**	**Subgroups**		***P*-value**
	Non-CVD	CVD	
	(n = 306)	(n = 37)	
Na^+^ (mmol/L)	141.1 ± 2.4	140.9 ± 2.6	0.317
K^+^ (mmol/L)	4.25 ± 0.37	4.34 ± 0.36	0.346
Ca^2+^ (mmol/L)	2.36 ± 0.11	2.37 ± 0.09	0.768
Mg^2+^ (mmol/L)	0.88 ± 0.08	0.85 ± 0.11	0.004
	Non-PAD	PAD	
	(n = 271)	(n = 15)	
Na^+^ (mmol/L)	141.1 ± 2.3	140.7 ± 3.2	0.094
K^+^ (mmol/L)	4.25 ± 0.37	4.21 ± 0.25	0.579
Ca^2+^ (mmol/L)	2.36 ± 0.10	2.33 ± 0.11	0.417
Mg^2+^ (mmol/L)	0.87 ± 0.08	0.85 ± 0.10	0.081
	Non-macrovascular complications	Macrovascular complications	
	(n = 243)	(n = 50)	
Na^+^ (mmol/L)	141.0 ± 2.3	141.0 ± 2.8	0.221
K^+^ (mmol/L)	4.24 ± 0.37	4.31 ± 0.34	0.555
Ca^2+^ (mmol/L)	2.36 ± 0.11	2.35 ± 0.10	0.667
Mg^2+^ (mmol/L)	0.87 ± 0.08	0.86 ± 0.10	0.017

Descriptive values are expressed as the mean ± standard deviation. Differences are assessed using ANCOVA, adjusted for age, gender, BMI, WC, hypertension and dyslipidemia.

*Abbreviations*: *CVD* Cardiovascular disease, *PAD* Peripheral artery disease, *Na* Sodium, *K* Potassium, *Ca* Calcium, *Mg* Magnesium.

## Discussion

Derangement of water and electrolyte balances may occur in subjects with DM, resulting from insulin deficiency, hyperglycemia and hyperketonemia [25]. The present study has determined that electrolyte levels are altered among Chinese subjects with different glucose tolerance statuses. Specifically, we verified that serum Mg^2+^ level was reduced in Chinese subjects with diabetic macrovascular complications.

### Alterations in serum electrolyte levels with elevated glucose levels

Significant reductions in serum Na^+^ and Mg^2+^ levels and an elevation in serum Ca^2+^ in subjects with DM were observed. These results were consistent with those reported by previous studies [6,7,26,27]. Furthermore, levels of serum Na^+^ and Mg^2+^ were negatively correlated with plasma glucose levels, measured as FPG, 2hPG and HbA1c, while Ca^2+^ level was positively correlated with HbA1c in subjects with DM.

Previous studies have estimated that serum Na^+^ concentrations decrease from 0.24 mmol/l to 0.29 mmol/l for every 1.0 mmol/l decrease in glucose concentration [28,29]. Our findings suggested there was a 0.32 mmol/l decrease of Na^+^ per 1.0 mmol/l fasting plasma glucose. Yajnik et al. observed that plasma Mg^2+^ concentrations were inversely related to plasma glucose levels in subjects with DM [30], and our results are in accordance with this.

In the present study, serum electrolyte levels of Na^+^ and Mg^2+^ decreased along with an elevation in the eGFR in subjects with DM. Under physiological conditions, most of the Na^+^ is reabsorbed in the proximal tubule of the kidney and Mg^2+^ is mostly reabsorbed in the loop of Henle [31]. Hyperglycemia-induced osmotic diuresis, which can increase excretion, is thought to be a primary mechanism underlying the decreased serum concentrations of Na^+^ and Mg^2+^ observed in response to elevated glucose levels. Additionally, overly aggressive volume re-expansion and glomerular hyperfiltration can induce renal Mg^2+^ wasting at the proximal tubule and the loop of Henle. Mg^2+^ reabsorption parallels Na^+^ reabsorption in the proximal tubules; therefore, volume expansion would further decrease the reabsorption of both Mg^2+^ and Na^+^[8,32].

In addition, we found that serum Ca^2+^ level was positively correlated with glucose level. This observation was consistent with previous studies [27,33,34]. Ca^2+^ is mainly reabsorbed in the proximal tubule. Its reabsorption is coupled to Na^+^ absorption, and it appears to compete with Mg^2+^ for transport in the loop of Henle. In the distal convoluted tubule, Ca^2+^ absorption is regulated independently of Na^+^, where numerous factors, such as calcitonin, parathyroid hormone, and vitamin D, can have marked effects on Ca^2+^ reabsorption and secretion [31,35]. Energy metabolism is associated with bone remodeling, and glucose levels also regulate parathyroid hormone and vitamin D levels [36]. This means that the excess excretion of Ca^2+^, induced by hyperglycemia, may be weakened by several pathways. Alternatively, the Resnick ionic hypothesis suggested that metabolic disorders, such as hypertension, metabolic syndrome, and diabetes, share a common, altered intracellular condition, characterized by decreased Mg^2+^ level and reciprocally elevated free intracellular Ca^2+^ level [37]. Intracellular Ca^2+^ plays a critical role in muscle contractions, insulin secretion, and glucose uptake after the binding of insulin to muscle cell membranes. The most common clinical manifestations of type 2 DM is insulin resistance, which mostly occurs in skeletal muscle, and the impairment of insulin secretion [38]. All these factors may account for the elevation of serum Ca^2+^ level in subjects with DM, and especially in patients whose eGFR levels are increased.

Furthermore, it has been observed that cellular membrane electrolyte transporter Na^+^-K^+^-ATPase dysfunction in diabetic subjects, can be secondary to hyperglycemia [39]. And the functions of Ca^2+^-Mg^2+^-ATPase, Na^+^/Ca^2+^ exchanger and Ca^2+^ pump, which are located in cell membrane, mitochondria or endoplasmic reticulum, have been shown to be impaired in diabetes [40,41]. Electrolyte disturbances within cells can be induced by them above mentioned. The combination of intracellular and extracellular electrolyte disturbances may be implicated in the pathogenesis of neuropathy, nephropathy and vascular complications in diabetic patients.

### Serum Mg^2+^ level is further decreased in subjects with diabetic macrovascular complications

We found that serum Mg^2+^ level was reduced in subjects with diabetic macrovascular complications. Ma et al. also demonstrated that, in African Americans and Caucasians, serum Mg^2+^ level was significantly lower in subjects with prevalent CVD and diabetes [42]. Recently, Larsson et al. performed a meta-analysis based on eight prospective studies and concluded that there was a significant inverse relationship between dietary Mg^2+^ intake and stroke risk [43]. It has also been observed that serum Mg^2+^ depletion is associated with some PAD symptoms, such as foot ulcers, in subjects with type 2 DM [44]. In addition, Resnick ionic hypothesis suggested that the intracellular Mg^2+^ level is generally suppressed, which summarizes the primacy of a common cellular defect in ion handling in various clinical manifestations of metabolic disorders [37]. Both decreased intracellular and extracellular Mg^2+^ concentrations may accelerate atherogenesis via the elevation of inflammatory cytokines and lipid oxidation, an increase in endothelial cell growth and the inhibition of cellular DNA repair [45-48].

Diabetic macrovascular complications, CVD in particular, are currently the leading cause of death for diabetic patients. It has been reported that even low Mg^2+^ levels that are still within the normal reference range are associated with all-cause mortality in patients with T2DM [10]. Our results would raise awareness of physicians, especially in China, and allow them to promptly correct mild electrolyte disturbances in diabetic patients. Even though many observational studies of electrolyte disturbances in patients with diabetes have consistently report that Mg^2+^ intervention may be extremely useful in the prevention and treatment of the cardiometabolic syndrome [49], some results of intervention studies, especially those involving daily electrolyte supplements for the prevention of DM or against diabetic complications, are controversial [50].

### Strengths and limitations

The first strength was that our subjects were from a large population-based survey. The symptoms and complications of the diabetes group were relatively mild, and the effects of iatrogenic factors on electrolytes were less than in those patients who were administrated in hospital. Secondly, we matched subjects among the NGR, IGR and DM groups to reduce the significant differences in gender and age distribution. A main limitation of our study was that we only measured the total serum electrolytes levels, but not free ionized or intracellular electrolytes or levels in other biological samples from the subjects [51], which need some special apparatus to measure [52]. Although questionnaire data are of great value in collecting the information of the history of non-fatal CVDs, misclassification remains a possibility [53]. Moreover, even though the analyses were adjusted for the common risk factors of age, gender, obesity, hypertension and dyslipidemia, unmeasured factors, such as eating habits and other medications, may have indirect effects on the electrolyte regulation and interfere with the relationship between CVD and electrolyte disturbances. Our study also did not elucidate the causal relationship of serum electrolyte levels on the development of DM and diabetic macrovascular complications.

## Conclusions

Our study advances our knowledge of electrolytes levels in Chinese subjects with diabetes that these subjects have decreased Na^+^ and Mg^2+^ and increased Ca^2+^ levels compared to normal subjects. These alterations in electrolyte levels may underlie many of the pathophysiologic and clinical characteristics of diabetes. Furthermore, significant negative associations were found between serum Na^+^, Mg^2+^ levels and glucose level respectively, while Ca^2+^ level was positively associated with glucose level. Of more importance, the subjects with lower serum Mg^2+^ level had an increased likelihood of diabetic macrovascular complications.

## Abbreviations

NGR: Normal glucose regulation; IGR: Impaired glucose regulation; DM: Diabetes mellitus; FPG: Fasting plasma glucose; 2hPG: Two hour post-load plasma glucose; HbA1c: Glycosylated hemoglobin A1c; ABI: Ankle brachial index; eGFR: estimated glomerular filtration rate; CVD: Cardiovascular disease; PAD: Peripheral arterial disease; Na+: Sodium; K+: Potassium; Ca2+: Calcium; Mg2+: Magnesium.

## Competing interests

The authors declare that they have no competing interests.

## Authors’ contributions

Shenqi Wang and Xuhong Hou performed the statistical analysis and wrote the manuscript; Yu Liu and Huijuan Lu participated in the data collection and checked the data; Li Wei contributed to discussion; Weiping Jia and Yuqian Bao participated in the design of this study and edited the manuscript. All authors have read and approved the final manuscript.
